# Pancreas volumes in humans from birth to age one hundred taking into account sex, obesity, and presence of type-2 diabetes

**DOI:** 10.1002/ca.20543

**Published:** 2007-09-18

**Authors:** Y Saisho, AE Butler, JJ Meier, T Monchamp, M Allen-Auerbach, RA Rizza, PC Butler

**Affiliations:** 1 Larry Hillblom Islet Research Center, UCLA David Geffen School of MedicineLos Angeles; 2 Division of Endocrinology, Diabetes and Hypertension, UCLA David Geffen School of MedicineLos Angeles; 3 Department of Molecular and Medical Pharmacology, UCLA David Geffen School of MedicineLos Angeles; 4 Division of Endocrinology, Diabetes, Metabolism and Nutrition, Mayo ClinicRochester, Minnesota

**Keywords:** computed tomography, histology, growth, aging, fat/parenchyma ratio

## Abstract

Our aims were (1) by computed tomography (CT) to establish a population database for pancreas volume (parenchyma and fat) from birth to age 100 years, (2) in adults, to establish the impact of gender, obesity, and the presence or absence of type-2 diabetes on pancreatic volume (parenchyma and fat), and (3) to confirm the latter histologically from pancreatic tissue obtained at autopsy with a particular emphasis on whether pancreatic fat is increased in type-2 diabetes. We measured pancreas volume in 135 children and 1,886 adults (1,721 nondiabetic and 165 with type-2 diabetes) with no history of pancreas disease who had undergone abdominal CT scan between 2003 and 2006. Pancreas volume was computed from the contour of the pancreas on each CT image. In addition to total pancreas volume, parenchymal volume, fat volume, and fat/parenchyma ratio (F/P ratio) were determined by CT density. We also quantified pancreatic fat in autopsy tissue of 47 adults (24 nondiabetic and 23 with type-2 diabetes). During childhood and adolescence, the volumes of total pancreas, pancreatic parenchyma, and fat increase linearly with age. From age 20–60 years, pancreas volume reaches a plateau (72.4 ± 25.8 cm^3^ total; 44.5 ± 16.5 cm^3^ parenchyma) and then declines thereafter. In adults, total (∼32%), parenchymal (∼13%), and fat (∼68%) volumes increase with obesity. Pancreatic fat content also increases with aging but is not further increased in type-2 diabetes. We provide lifelong population data for total pancreatic, parenchymal, and fat volumes in humans. Although pancreatic fat increases with aging and obesity, it is not increased in type-2 diabetes. Clin. Anat. 20:933–942, 2007. © 2007 Wiley-Liss, Inc.

## INTRODUCTION

β-cell mass cannot be quantified yet in humans in vivo, and so studies of human β-cell mass have relied primarily on autopsy tissue. There are several limitations to this approach. At autopsy, typically, the whole pancreas is not removed so that only a sample of pancreas is available. From this sample, after fixation and embedding, sections are cut to permit immunohistochemical staining for insulin, which provides the fractional area of β-cells. Since fat is removed from the pancreas during this process, the β-cell fractional area identified by this approach is the fraction of the fat-free pancreas (acinar tissue) occupied by insulin positive cells. Our primary goal in this study was to obtain population data for pancreas acinar volumes at different ages in humans to permit calculation of β-cell mass from fractional β-cell/acinar pancreatic areas in humans.

The available data suggest that there are effects of age and adiposity on pancreas volume. For example, anatomical studies report decreased pancreas volume with aging in humans (Rossle, 1921; Tanaka et al., 1989; Ogiu et al., 1997) and histological studies report atrophy, fibrosis, and fatty infiltration of the pancreas in the aging population (Noronha et al., 1981; Stirling, 1994). Other anatomical studies report effects of height, weight, and body mass index (BMI) on pancreas volume (Kloppel et al., 1985; Ogiu et al., 1997; de la Grandmaison et al., 2001). Kloppel et al. (1985) report that the pancreatic volume is increased in obese versus lean subjects.

Although these data point to likely changes in pancreas acinar volumes with aging and in response to obesity, there is insufficient data to permit the development of population curves that could be used for the calculation of β-cell mass in humans from autopsy samples. Recent advances in computed tomography (CT) permit accurate noninvasive measurement of organ volume (Disler et al., 1994; Nawaratne et al., 1997; Sandrasegaran et al., 1999; Geraghty et al., 2004). Moreover, this approach permits the measurement of the proportion of the organ occupied by fat. Some investigators believe that pancreatic fat may have deleterious effects on pancreatic β-cells (Unger and Orci, 2001; Assimacopoulos-Jeannet, 2004). It is as yet unclear if pancreatic fat is increased in people with diabetes compared to BMI matched nondiabetic controls. Therefore, in this study, we also sought to establish population curves for pancreas volume (total, and relative contributions of fat and parenchymal tissue) in humans taking into account the effects of gender and obesity by use of CT scan in a large population from birth to age 100 years. In addition, we sought to establish if pancreatic fat is increased in people with type–2 diabetes, and the impact of type–2 diabetes on pancreatic parenchymal volume.

## METHODS

### Subjects

UCLA institutional review board permission (IRB No. G05-10-113-02 and No. 06-05-046-02) was obtained for these studies. To study the pancreas volume in childhood and adolescence, a total of 135 (male 77, female 58) subjects aged 20 years and under were evaluated. Patients who had undergone a clinical abdominal CT scan at UCLA between 2003 and 2006 were chosen at random from the UCLA Department of Radiology database. To establish pancreas volumes in adults, pancreas CT scans from 1721 adult subjects (male 724, female 997) over 20 years of age who had undergone a whole body PET/CT at UCLA in 2005 or 2006 were evaluated. To examine pancreas volume in diabetic subjects, 165 type-2 diabetic adults (male 76, female 89; mean age 66 ± 11 years) were evaluated. Information regarding age, sex, height, weight, and presence of diabetes was obtained from the UCLA Department of Nuclear Medicine PET/CT clinical database. The races of the adult subjects were as follows: Caucasian (74%), Hispanic (9%), Asian (11%), African-American (5%), and others (1%). In light of the relatively small proportion of non-Caucasian subjects in these studies, it is not possible to make meaningful comparisons between races in the measured parameters.

Cases were excluded if they had any abdominal condition potentially affecting pancreas morphology (e.g., pancreatitis and peritonitis) or if precise delineation of the pancreas from adjacent structures was not possible. In all cases, the abdominal CT was evaluated by an independent radiologist to con.rm the absence of pancreas pathology.

### Computed Tomography

#### Subjects from birth to age 20 years

CT images were acquired with standard clinical abdominal CT protocol utilizing a multidetector Somatom Sensation 16 CT scanner (Siemens Medical Systems, Iselin, NJ). Patients were administered oral contrast (barium sulfate). After an infusion of intravenous contrast (2 ml/kg body weight of Omnipaque), contiguous 2.5-or5-mm axial images of the abdomen and pelvis were obtained. The CT parameters were as follows: 120 kVp, 55 mAs, 0.5-sec tube rotation, 1.5-mm slice collimation, and a table speed of 27.6-mm per rotation (pitch, 1.15). The images were transferred to a PC workstation and analyzed using Vitrea 2 software (version 3.5, Vital Images, Minnetonka, MN).

#### Subjects from age 20–100

CT images were acquired as part of PET/CT Imaging using the Reveal RT Scanner. This system combines a dual slice detector Somatom Emotion CT scanner (Siemens Medical Systems) with an ECAT ACCEL PET scanner (Siemens/CPS Innovations, Knoxville, TN). Patients were asked to drink 900 ml of barium sulfate. A whole body CT scan with intravenous contrast (110 ml of Omnipaque at a rate of 1.2 ml/sec with 54 sec delay) from the base of the skull to the mid-thighs was performed. The CT parameters were as follows: 130 kVp, 120 mAs, 1-sec tube rotation, 4-mm slice collimation, and a table speed of 8 mm/rotation (pitch, 1.0). CT images were reconstructed using conventional filtered back projection at 5-or6-mm axial intervals. The images were transferred to a PC workstation and analyzed using Vitrea 2 software (version 3.5, Vital Images).

### Phantom Study

**Fig. 1:**
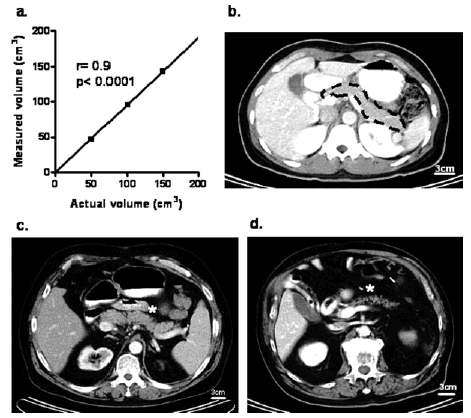
Phantom control and representative CT images demonstrating the pancreas in normal, obese, and elderly individuals. **a:** Phantom study of volume measurement (SD at 50 = 0.8, at 100 = 0.5, and at 150 = 1.2). **b:** Representative CT image of a normal pancreas (33-year-old female, BMI 24.1). Pancreas is indicated by the broken line. **c:** Representative CT image of an obese subject (53-year-old male, BMI 35.3) showing an enlarged pancreas. CT density of the pancreas is low and interlobular fissures are seen on the surface edge. **d:** Representative CT image of an elderly subject (83-year-old male, BMI 25.8). The pancreas is atrophic. CT density is decreased and inhomogeneous with marked interlobular fissures present.

To validate the accuracy of volume measurement, we performed a phantom study using balloons filled with tap water. The known volume of 50, 100, and 150 ml was scanned by the same PET/CT protocol and measured for each volume by Vitrea 2 software. The measured volume showed a close relationship with actual volume (r = 0.9, P < 0.0001; Fig. 1a).

### Measurement of Pancreas Volume

All images were analyzed at a window level of 40 Houns-field units (HU) and window width of 300 HU. Pancreata were identified based on the typical landmarks (splenic vein, superior mesenteric artery) as previously described (Geraghty et al., 2004). Using the hand-outlined pancreas, we first measured the total pancreas volume by use of the surface tool in Vitrea 2. This outlined pancreas volume inevitably contains fat, most of which in the case of the pancreas is within interlobular adipocytes, but also is present in occasional groups of perivascular adipoctyes within the pancreas. A lesser but ill-defined amount of fat is present within acinar cells. To establish a best possible measure of the pancreas volume occupied by fat versus acinar tissue, we then employed an approach that depends on the differential density of fat versus acinar tissue by CT. This approach has been used previously to derive fat content in other organs, for example, the liver in which case most of the fat is intracellular (Kodama et al., 2007). Using the same outlined region, but a different imaging software tool (Vitrea 2 sculpt tool), we now identified the pancreas volume free of fat. This was accomplished by using a window level of +40 HU (recommended range for pancreas parenchymal tissue is from +30 to +50 HU) in contrast to that for fat, which is in the negative range (from −50 to −150 HU) (Wegener, 1993).

By this approach, the total and parenchymal pancreas area (cm^2^) on each image was computed as the actual area or the respective pixel area, which is the product of the number of pixels in each outline, respectively. Pancreas volume per section was calculated as the product of each pancreas area and the CT section thickness. Total and parenchymal volume of pancreas was computed by summing up the volume from each section that included a piece of pancreas tissue. Pancreas fat tissue volume was calculated as total pancreas volume—parenchymal pancreas volume (cm^3^). It should be emphasized here that since the pancreas fat volumes assume that the measured fat within the pancreas was in adipocytes to the extent that this is in error (i.e., is in parenchymal cells), the measured fat volume will be artificially increased. The measurement of pancreas volume was carried out by a single investigator (Y.S.). Intraobserver CV of pancreas volume (computed in five cases studied on five occasions) was (mean) 2.3% (range) 0.8%−3.5%.

### Histological Evaluation of Parenchymal Fat Accumulation

Parenchymal fat accumulation was also evaluated histologically. Tissue was obtained at autopsy from the tail of the pancreas. Potential cases were identified by retrospective analysis of the Mayo Clinic autopsy database. Cases were excluded if pancreatic tissue had undergone autolysis or showed evidence of acute pancreatitis. Age-matched lean and obese subjects with or without type-2 diabetes were evaluated (male 28, female 19, age 75 ± 8 years). Patients' characteristics of each group are shown in Table 1. To examine the effect of aging, middle-aged lean, and obese nondiabetic subjects (male 10, female 8, age 50 ± 2 years) were also evaluated.

**TABLE 1:** Characteristics of Patients From Whom Pancreata Were Harvested at Autopsy

	LND	LD	OND	OD
*n*(M/F)	11(7/4)	10(6/4)	13(6/7)	13(9/4)
Age (years)	74 ±11	77 ±7	75 ±6	74 ±7
BMI (kg/m^2^)	22.6±2.2	22.9±2.1	35.6±7.6*	34.6±5.5#

Values are means ±SD

LND, lean nondiabetic; LD, lean diabetic; OND, obese nondiabetic; OD, odese diabetic

* *P*>0.01 vs.LND

# *P*>0.01 vs.LD

Each pancreas was fixed in formaldehyde and embedded in paraffin for subsequent analysis as previously described (Butler et al., 2003). Thick sections (5 μm) of pancreas tissue were stained with hematoxylin-eosin, and the entire pancreatic section was imaged at 40× magnification (4× objective). Since pancreatic fat is extracted during specimen dehydration, intrapancreatic fat appears in histologic sections as lobulated spaces within the pancreas. These intrapancreatic fat spaces were evaluated by morphometric analysis using Image Pro Plus software (Media Cybernetics, Silver Springs, MD). Total pancreatic exocrine and endocrine area, including intrapancreatic fat spaces, were measured (excluding any fat between lobules or adherent to the exterior of the pancreas). The intrapancreatic fat deposits were then evaluated (i.e., those within the exocrine tissue), and the ratio of pancreatic fat to total pancreatic area was calculated. This data is presented as a percentage and is analogous to the fat/parenchyma ratio calculated from CT studies. To the extent that the fat and acinar tissue proportions differ in the different regions of the pancreas, the availability of tail of pancreas only is a limitation of these studies.

### Statistical Analysis

Subject characteristics and results are reported as mean ± SD. Results in figures are presented as mean ± SEM. All statistical analyses were performed using the StatView program for Windows (SAS Institute, Cary, NC). Analysis of variance (ANOVA) followed by Fisher's LSD was used for post hoc analysis. A *P*-value < 0.05 was considered statistically significant.

## RESULTS

### CT Imaging of Pancreata

Pancreas volume was measured by contouring the surface edge of the pancreas in each CT slice as shown in Figure 1b. CT density of the pancreas varied widely, even within the same pancreas. In obese and older subjects, the CT density of the pancreas tended to be lower, consistent with the accumulation of pancreatic fat (Fig. 1c and 1d).

### Pancreas Volume in Childhood and Adolescence

#### Growth of the pancreas

**Fig. 2:**
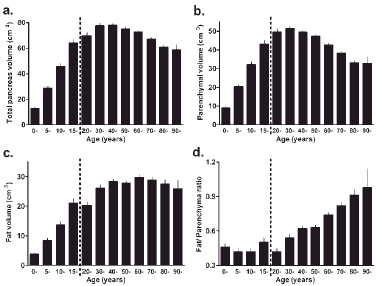
Total, parenchymal, and pancreatic fat volumes with age calculated from CT images. **a:** Total pancreas volume. **b:** Parenchymal volume. **c:** Fat volume. **d:** Fat/parenchyma ratio (F/P ratio). Total pancreas volume increases with age until ∼30 years and then reaches a plateau. After age 60, total pancreas volume declines due to a decline in parenchymal pancreas volume. Pancreatic fat remains proportionate to pancreas parenchyma until age ∼50 years, after which the F/P ratio increases because of loss of pancreas parenchyma, but not fat. For *n* value for each age group, see Supplementary Table 2.

Pancreas volume in childhood and adolescence (under 21 years) is shown in Figure 2 (also see Supplementary Table 1 and Supplementary Figs. 1 and 2). Total pancreas volume linearly increased with age (*r* = 0.9, *P* < 0.0001). Similarly, parenchymal volume also increased in a linear manner with age (*r* = 0.9, *P* < 0.0001). Approximately 80% of both total and parenchymal volume were explained by the relationship with age (*r*2 = 0.79 and 0.80, respectively), and these relationships were expressed by the following equations:

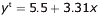
(1)

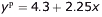
(2)
where *y*^t^ is the total pancreas volume (cm^3^), *y*^p^ the parenchymal pancreas volume (cm^3^), and *x* the age (years). The data for each individual are shown in Supplementary Figures 1 and 2, showing that the variance of individuals from this relationship was modest until toward adulthood when the variance increases.

The pancreatic fat volume increased throughout childhood (*r* = 0.8, *P* < 0.0001) in proportion to total pancreas growth, so that the ratio of pancreatic fat/parenchyma (F/P ratio) remained constant (Figs. 2c and 2d and Supplementary Table 1). These changes in pancreas volume in childhood were comparable in both genders, although after 10 years of age, total, and parenchymal volumes in females tended to be smaller than in males (Supplementary Table 1), consistent with the less overall somatic growth.

### Pancreas Volumes in Adults

Patient demographics and pancreas volume data are summarized in Table 2 and in Figure 2 (also see Supplementary Table 2 and Supplementary Figs. 1 and 2).

**TABLE 2:** Characteristics of Adult Subjects Involved in CT Imaging Studies of the Pancreas

	Total subjects	Male	Female
*n*	1721	724	997
Age (years)	58±16	59±16	57±15
BMI(kg/m^2^)	25.4±4.8	26.0±4.2	25.0±5.2*
Pancreas volume Total (cm^3^)	72.4±25.8	85.2±26.9	63.0±20.5*
Parenchyma (cm^3^)	44.5±16.5	48.8±17.5	41.3±15.0*
Fat(cm^3^)	27.9±15.7	36.4±17.0	21.7±11.2*
Fat/parenchyma ratio	0.69±0.44	0.83±0.46	0.59±0.39*

Values are means ±SD

* *P*<0.01 vs.male

#### Effect of age on pancreas volume

After increasing relatively rapidly in childhood, the total pancreas volume changes little during age 20–60 years and then declines thereafter (Fig. 2a; Supplementary Table 2 and Supplementary Fig. 1). Parenchymal pancreatic volume reaches a maximum in the third decade in both males (56.4 cm^3^) and females (48.3 cm^3^), remaining constant until ∼60 years of age. Thereafter, parenchymal volume gradually decreases in both the genders (Fig. 2b; Supplementary Table 2 and Supplementary Fig. 2).

As shown in Figure 2 and Supplementary Figures 1 and 2, the decline in pancreas volume after age 60 was best described by the following equations.

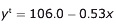
(3)

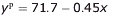
(4)
where *y*^t^ is the total pancreas volume (cm^3^), *y*^p^ the parenchymal pancreas volume (cm^3^), and *x* the age (years). The data for each individual are shown in supplementary Figures 1 and 2, showing that the variance of individuals from this relationship is much greater than in childhood, so that the studies using mean computed pancreas volumes need to have a sufficiently large number of individuals to justify this assumption.

Pancreatic fat volume increases with age in adults (Fig. 2c and Supplementary Table 2). In males, pancreatic fat volumes increase in the third and fourth decade and then remain constant until the seventh decade (Supplementary Table 2). In contrast, in females, pancreatic fat volumes remain remarkably unchanged throughout the second to ninth decade. As a consequence, while the F/P ratio increases with aging in both men and women, it does so to a greater extent in men with the composite effects of decreased parenchymal and increased fat volume (Supplementary Table 2).

### Effect of BMI on Pancreas Volume

**Fig. 3:**
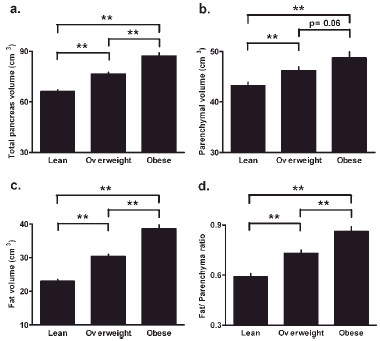
Effect of BMI on pancreatic volume. Total pancreatic volume (**a**), parenchymal volume (**b**), fat volume (**c**), and fat/parenchyma ratios (F/P ratio) (**d**) were measured from CT scan images in lean (*n* = 460), overweight (*n* = 460), and obese groups (*n* = 230). All components of pancreatic volume are significantly increased in overweight and obese subjects compared with lean subjects. **P < 0.01.

To examine the effect of BMI on pancreatic volume, we classified the subjects into lean (BMI < 25 kg/m^2^), overweight (25 kg/m^2^ ≤ BMI < 30 kg/m^2^), and obese (BMI ≥ 30 kg/m^2^) groups (Fig. 3 and Supplementary Table 3). Sexand age-matched subjects were then identi.ed (lean *n* = 460, overweight *n* = 460, obese *n* = 230) for each group with a resulting mean BMI of 22.1 ± 1.8, 27.1 ± 1.4, and 33.6 ± 3.4 kg/m^2^, respectively. Height was comparable in the three groups (169 ± 10, 169 ± 9, and 169 ± 11 cm in lean, overweight, and obese groups, respectively; *P* = 0.6).

Total pancreatic volume was 16 and 32% greater in the overweight and obese groups compared with the lean group (66.2 ± 22.0, 76.6 ± 23.8, and 87.3 ± 30.3 cm^3^ in lean, overweight, and obese groups, respectively). Pancreas fat volume was 32 and 68% greater in the overweight and obese groups compared with the lean group (23.0 ± 12.4, 30.4 ± 14.3, and 38.6 ± 18.9 cm^3^ in lean, overweight, and obese subjects, respectively). Parenchymal volume was increased to 7 and 13% in the overweight and obese groups compared with the lean group (43.2 ± 15.7, 46.2 ± 16.2, and 48.7 ± 18.0 cm^3^ in lean, overweight, and obese subjects, respectively). The effect of obesity on pancreatic volumes was comparable in both genders. Given the greater increment in pancreatic fat versus parenchyma in obesity, the pancreatic F/P ratio was increased in overweight and obese groups compared with the lean group (0.59 ± 0.37, 0.73 ± 0.45, and 0.86 ± 0.44 in lean, overweight, and obese groups, respectively) (Fig. 3d and Supplementary Table 3).

**Fig. 4:**
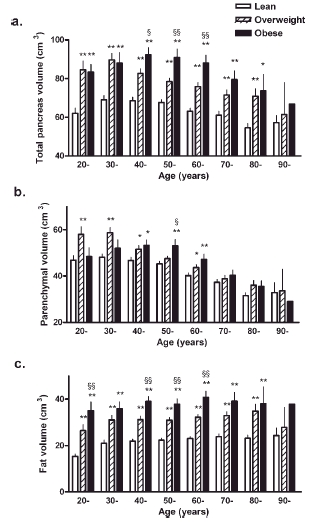
Interaction of aging and BMI on pancreatic volumes. Total pancreas volume (**a**), parenchymal volume (**b**), and fat volume (**c**) are shown for each decile for lean, overweight, and obese groups. *P < 0.05, **P < 0.01 vs. lean. §*P* < 0.05, §§*P* < 0.01 vs. overweight.

The interaction of age and BMI on pancreas volume is shown in Figure 4. Total pancreas and fat volume were increased in overweight and obese subjects compared with the lean subjects in every decile, although the difference did not reach significance among individuals in their 90s (Fig. 4a and 4c). The effect of obesity on parenchymal volume is apparent until the seventh decade (Fig. 4b).

Pancreatic volume remained constant in adults from 20 to 60 years of age (Figs. 2a and 2b and Supplementary Figs. 1 and 2). Taking into account the effects of obesity on pancreatic volume, there was a significant correlation between pancreas volume and BMI (*r* = 0.4, *P* < 0.0001 and *r* = 0.2, *P* < 0.0001 for total and parenchymal volume, respectively). This increase in pancreatic volume with BMI was best expressed by the following equations:

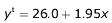
(5)

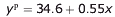
(6)
where *y*^t^ is the total pancreas volume (cm^3^), *y*^p^ the parenchymal pancreas volume (cm^3^), and *x* the BMI. These equations suggest that the total and parenchymal pancreas volumes are increased by about 10 cm^3^ and 3 cm^3^, respectively, with each 5 kg/m^2^ increase in BMI, although it should be noted that there is a considerable variation between the individuals with respect to pancreas volume and that these relationships might only be reasonably applied in relatively large population studies.

### Effect of type–2 diabetes on pancreas volume

**Fig. 5:**
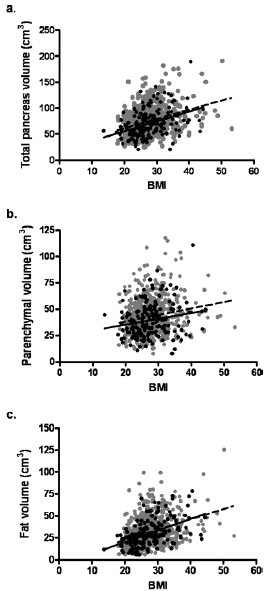
Relationship between BMI and pancreatic volumes. Despite considerable individual variance there is a significant relationship between BMI and total (**a**), parenchymal (**b**), and fat (**c**) volumes in nondiabetic subjects (gray dots, broken line) and subjects with type-2 diabetes (solid dots and solid line). The relationships are comparable in individuals with type-2 diabetes (T2DM) and nondiabetics (ND). (a) ND *r* = 0.3, *P* < 0.0001; T2DM *r* = 0.4, *P* < 0.0001, (b) ND *r* = 0.2, *P* < 0.0001; T2DM *r* = 0.2, *P* = 0.01, (c) ND *r* = 0.4, *P* < 0.0001: T2DM *r* = 0.5, *P* < 0.0001.

By CT scan and analysis, total and parenchymal pancreas volumes are decreased in subjects with type-2 diabetes compared with nondiabetic subjects (70.0 ± 26.5 vs. 74.9 ± 27.0 cm^3^, *P* < 0.05; and 39.7 ± 16.4 vs. 43.1 ± 16.5 cm^3^, *P* < 0.05, total and parenchymal volumes, respectively). However, there is no difference in fat volume between subjects with type–2 diabetes compared with the nondiabetic subjects (Table 3). Consistent with these findings, the relationship between pancreas volume and BMI are similar in diabetic and nondiabetic subjects (Fig. 5a–c).

**TABLE 3:** Physical and Pancreatic Characteristics of Nondiabetic and Diabetic Subjects

	Nondiabetic	diabetic
*n*(M/F)	660(304/356)	165(76/89)
Age(years)	66±12	66±11
BMI(kg/m^2^)	27.6±5.0	27.7±5.6
Pancreas volume Total(cm^3^)	74.9±27.0	70.0±26.5*
Parenchyma(cm^3^)	43.1±16.5	39.7±16.4*
Fat(cm^3^)	31.7±16.6	30.3±15.2
Fat/parenchyma ratio	0.81±0.50	0.85±0.48

Values are means ±SD

* *P*<0.05 vs.nondiabetic

**Fig. 6:**
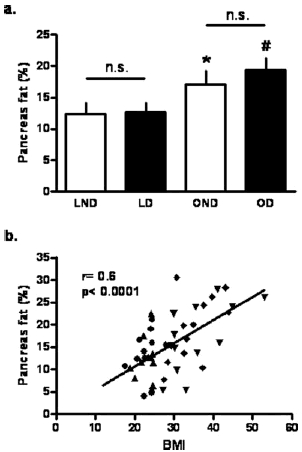
Comparison of pancreatic fat percentages from pancreata obtained at autopsy in lean and obese subjects with or without type-2 diabetes. **a:** Histological comparison of pancreas fat (%) between lean and obese subjects with or without type-2 diabetes. LND, lean nondiabetic; LD, lean diabetic; OND, obese nondiabetic; OD, obese diabetic. *P < 0.05 vs. LND. #*P* < 0.05 vs. LD. **b:** Correlation analysis between BMI and histological pancreas fat (%). Circle, LND; triangle, LD; reverse triangle, OND; rhombus, OD.

Morphometric analysis of autopsied pancreata showed that the pancreatic fat was also comparable between individuals with type-2 diabetes and nondiabetic controls (Fig. 6). However, in nondiabetic individuals, pancreas fat increased with obesity (*r* = 0.6, *P* < 0.0001) and age (*r* = 0.7, *P* < 0.0001), both consistent with CT scan studies (vida supra).

## DISCUSSION

We have established population data for pancreas volumes from birth to age 100 years in humans. A variety of prior studies have examined changes in pancreatic volume by use of autopsy or imaging approaches (Table 4). In this study, we have extended those findings in the following ways. By the use of the property of differential densities of fat and parenchymal tissue observed by CT, we further documented the relative contributions of fat versus parenchymal tissue to total pancreas volume in humans taking into account the effects of age, sex, and BMI. We corroborated these findings by the examination of pancreata at autopsy in a smaller group (*N* = 47) of adult subjects. Furthermore, we examined the effect of type-2 diabetes on these parameters (both by CT scan and autopsy studies).

**TABLE 4:** Summary of Literature Reports on Pancreas Weight/Volume in Humans

					Pancreas weight(g) or volume(cm^3^)	
					
	*n*M/F	Race	Age(years)	BMI(kg/m^2^)	Total	Parenchyma
Anatomical studies Rossle et al.(1921)	46/30	N/A	41–50	N/A	M 72.4g	N/A
					F 66.9g	
Westermark et al.(1978)	8/7	N/A	75/8	N/A	76/6 cm^3^	62±5 cm^3^
Rahier et al.(1983)	4/4	N/A	54	N/A	82 g	N/A
Kloppel et al.(1985)	Lean 4/3	N/A	54±14	20.3±4.6	69±19 cm^3^	40±11 cm^3^
	Obese 3/1		58±18	29.0±4.6	135±17 cm^3^	80±28 cm^3^
Lohr et al.(1987)	19	N/A	40±18	N/A	86.5±33.0 cm^3^	71.9±25.9 cm^3^
Clark et al.(1988)	4/6	N/A	67±8	N/A	119±32g	N/A
Tanaka et al.(1989)	2313/538	Asian	20–50	N/A	M 135.1±37.8 g	N/A
					F 112.2±30.4g	
Ogiu et al.(1997)	262/99	Asian	45–49	N/A	M 103.9±28.1 g	N/A
					F 95.2±26.2	
de la Grandmaison	355/329	Caucasian	M 42±17	M 22.8±3.3	M 144±39g	N/A
et al.(2001)			F 49±20	F 22.5±4.5	F 122±35g	
Sakuraba et al.(2002)	10/5	Asian	52±11	21.3±2.7	122±29 g	N/A
Yoon et al.(2003) Imaging studies CT	6/3	Asian	41±14	23.8±1.9	77.1±14.6 g	N/A
Goda et.al(2001)	10/12	Asian	46±6	N/A	71.5±18.7 cm^3^	N/A
Geraghty et al.(2001)			F 49±18		F 64.4±18.1 cm^3^	
*This study*	724/997	*Mixed*	M 58±16	M 25.4±4.8	m 85.2±26.9 cm^3^	M 48.8±17.5 cm^3^
MRI Williams et al.(2007)	12/0	N/A	29	25	101.0±19.5 cm^3^	N/A

Values are means ±SD

M, male;F, female.

We report that the pancreas volume increases in a linear fashion from birth to age 20 years and thereafter reaches a plateau, a finding consistent with available anatomical studies (Rossle, 1921; Tanaka et al., 1989; Ogiu et al., 1997). Before age 20, ∼80% of total and parenchymal pancreas volume can be explained by age in a simple regression analysis. Thus, parenchymal pancreas mass during childhood mirrors somatic growth (Altman, 1962; Valentin, 2002). Although pancreatic fat volume increases with age, it does so in relation with parenchymal tissue so that the F/P ratio remains constant from birth to age 20 years.

From age 20 to 60 years, mean pancreas volumes are relatively constant in humans but with considerable between subject variance. We report that the pancreas volumes are greater in males than females, which is consistent with the anatomical studies (Rossle, 1921; Tanaka et al., 1989; Ogiu et al., 1997; de la Grandmaison et al., 2001). The mean total pancreas volume in adults reported here (85.2 ± 26.9 cm^3^ in males and 63.0 ± 20.5 cm^3^ in females) is in close agreement with prior CT studies. For example, Geraghty et al. (2004) reported that the pancreas volume was 87.4 ± 21.3 cm^3^ in males and 64.4 ± 18.1 cm^3^ in females. Goda et al. (2001) also reported that the mean pancreas volume in healthy subjects was 71.5 ± 18.7 cm^3^. Pancreas volumes measured by CT scan are comparable to some, but lower than other anatomical studies of pancreatic mass (see Table 4 for summary of studies). One possible explanation for the larger pancreatic volume/mass from anatomical studies versus imaging studies is the difficulty of removing all the fat surrounding the pancreas during dissection, a task made more difficult by the fact that fat and pancreatic tissue have a comparable texture and color on dissection.

We also report that in adults past 60 years of age, both total and parenchymal pancreas volumes gradually decline. Atrophy and fat infiltration of the pancreas have been reported with aging in humans (Noronha et al., 1981; Stirling, 1994), and previous anatomical studies reported that pancreas weight decreased after age 60 (Rossle, 1921; Tanaka et al., 1989; Ogiu et al., 1997). Ultrasound studies report increased pancreatic echogenicity with aging, suggesting the age–related fat accumulation in the pancreas (Worthen and Beabeau, 1982; Glaser and Stienecker, 2000). Others have reported age–related morphological changes of the pancreas by CT imaging (Heuck et al., 1987; Kreel et al., 1977). Heuck et al. (1987) reported the decreased pancreatic volume and increased lobulation in elderly humans. We extend these findings to report that it is the parenchymal component of the pancreas (and not pancreatic fat) that declines with aging. We also report that the pancreatic fat volume increases until the third decade but thereafter remains constant. Consequently, the F/P ratio increases in both the genders.

Our study also examined the effect of BMI on pancreas volume in adults. Although organ size usually correlates with body height and weight (Ogiu et al., 1997; de la Grandmaison et al., 2001), the relationship between pancreas size and BMI has not yet been elucidated. Kloppel et al. (1985) reported the pancreatic volume in obese subjects to be double that of lean subjects in 11 autopsies. In another autopsy study, it was concluded that the pancreas volume increases with BMI in males, but not in females. In females, pancreas volume was related instead of height (de la Grandmaison et al., 2001). Silva et al. (1993) reported that the diameters of the head, body, and tail of the pancreas measured by ultrasonography are related to BMI. Others reported that the pancreatic echogenicity increases with BMI, implying fat accumulation in the pancreas of obese subjects (Worthen and Beabeau, 1982). In this study, we report that the total pancreas volume (∼30%) and pancreas parenchymal volume (∼10%) increase in response to obesity in both genders. We also report that the accumulation of fat in the pancreas is increased (∼70%) in obese versus lean subjects.

Finally, we report the effect of type-2 diabetes on pancreatic volume. Total and parenchymal pancreas volumes were slightly but significantly (∼7% and ∼8%, respectively) decreased in diabetic subjects when compared with those of the nondiabetic subjects. Pancreas atrophy in type-1 diabetic subjects has been reported in anatomical and histological studies (Foulis and Stewart, 1984; Lohr and Kloppel, 1987). Imaging studies are consistent with those findings (Fonseca et al., 1985; Gilbeau et al., 1992; Alzaid et al., 1993; Silva et al., 1993; Chiarelli et al., 1995; d'Annunzio et al., 1996; Altobelli et al., 1998; Goda et al., 2001; Williams et al., 2007). Goda et al. (2001) reported a decrease in pancreas volume in type-1 diabetes measured by the CT scan. Measurement of pancreas volume in type-2 diabetes has been reported to be decreased (Fonseca et al., 1985; Kloppel et al., 1985; Migdalis et al., 1991; Gilbeau et al., 1992; Alzaid et al., 1993) or no different to controls (Westermark and Wilander, 1978; Clark et al., 1988; Silva et al., 1993; Goda et al., 2001). The present studies in a large age, sex, and BMI-matched cohort identified a decreased parenchymal volume in type-2 diabetes when compared with that of nondiabetic controls.

A secondary objective of the present studies was to address the question, is pancreatic fat increased in type-2 diabetes? We found no increase in pancreatic fat in type-2 diabetes whether measured by a CT-imaging-based approach or by histology at autopsy. A limitation of the latter approach was that the histological measurement of fat was indirect since fat is removed during tissue processing and section staining. Measurement of pancreatic fat by CT scan imaging is also by necessity indirect, and unable to distinguish between fat in adipocytes and intracellular fat in parenchymal cells. These limitations not withstanding, the results of the evaluation of measured pancreatic fat by these two approaches showed the same pattern, that is, increasing fat with BMI but no difference between diabetic and nondiabetics. These findings are consistent with the limited available histological (Clark et al., 1988) and imaging (Gilbeau et al., 1992) data that found no difference in pancreatic fat in type-2 diabetes when compared with nondiabetic controls.

In conclusion, we have established a population data for total and parenchymal pancreatic volumes in humans by the use of CT scan imaging. We also report the relative contributions of fat and parenchymal tissue to total pancreas volume throughout life, with the effects of obesity (increased pancreatic fat) and type-2 diabetes (no effect on pancreatic fat). These data will be of use in future studies seeking to examine β-cell mass (versus cross-sectional β-cell area) in humans of different ages, BMI, and with or without type-2 diabetes. These data are also unique in as much as they provide the first comprehensive report that pancreatic fat is not increased in humans with type-2 diabetes.
