# Supplementary material for: Pancreas volumes in humans from birth to age one hundred taking into account sex, obesity, and presence of type-2 diabetes
Source: Clin Anat. 2007 Sep 18;20(8):933–42. doi: 10.1002/ca.20543 (PMC2680737; doi:10.1002/ca.20543)
Supplement: Supplementary file 1 [file ca0020-0933-SD1.pdf]

Appendix for Online Publication

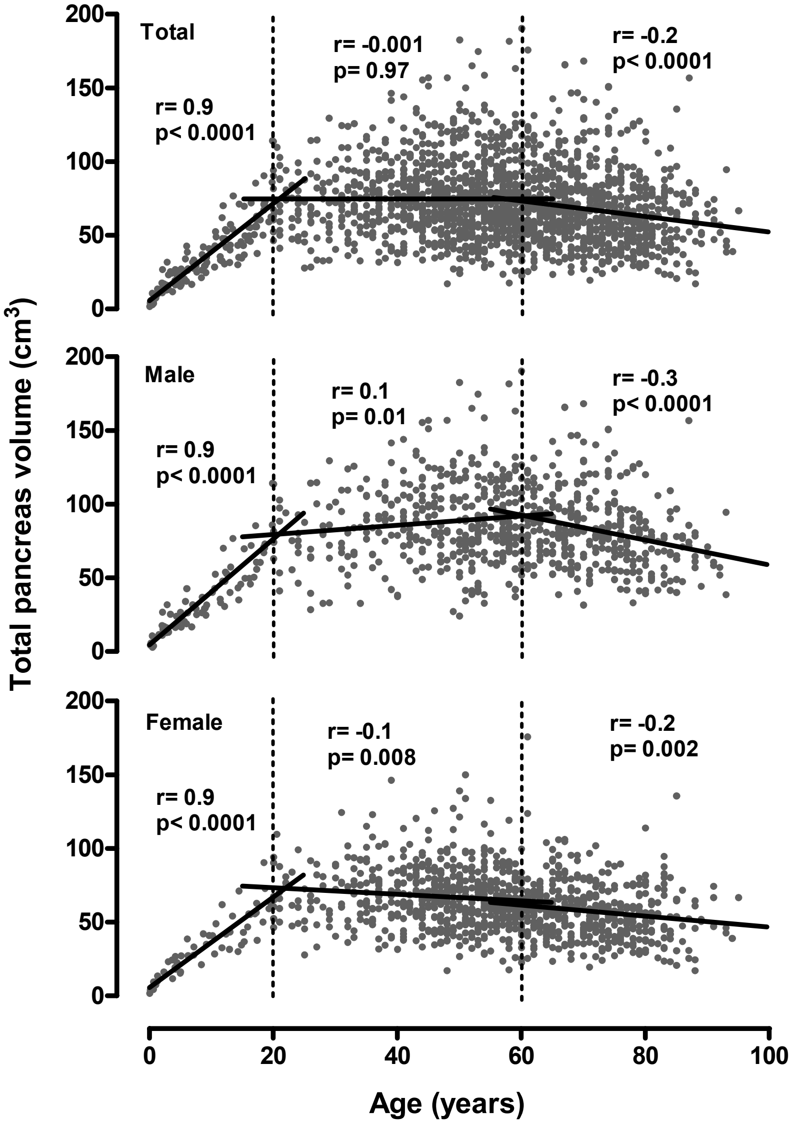

Supplementary Figure 1. Distribution of total pancreas volume from birth to age 100 years. There is a strong correlation between age and total pancreas volume from birth to age 20 years (y (volume; cm^3) = 5.5 + 3.31x (age; years), y= 4.5 + 3.60x, and y= 6.2 + 3.06x in total, male, and female groups, respectively). There is a negative correlation between age and total pancreas volume after age 60 years (y= 106.0 − 0.53x, y= 143.4 − 0.85x, and y= 83.1 − 0.37x in total, male, and female groups, respectively). There is no marked correlation between age and total pancreas volume from age 20 to 60 years.

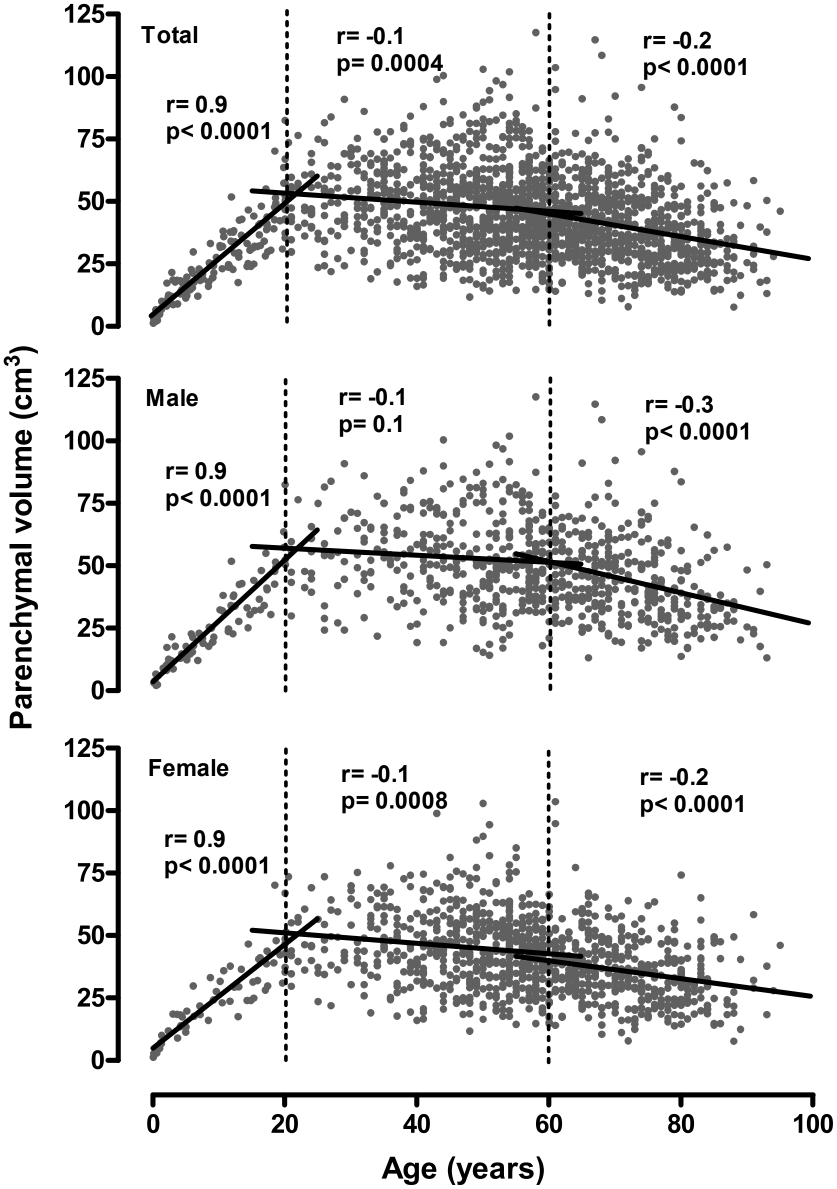

Supplementary Figure 2. **Distribution of parenchymal volume from birth to age 100 years. There is a strong correlation between age and parenchymal volume from birth to age 20 years (y (volume; cm^3) = 4.3 + 2.25x (age; years), y= 3.4 + 2.44x, and y= 4.9 + 2.08x in total, male, and female groups, respectively). There is a negative correlation between age and parenchymal volume after age 60 years (y= 71.7 − 0.45x, y= 88.7 − 0.62x, and y= 60.4 − 0.35x in total, male, and female groups, respectively). There is significant, but weak negative correlation between age and parenchymal volume from age 20 to 60 years.**

Supplementary Table 1. **Growth of Pancreas in Childhood and Adolescence**

Age (years)	n	Pancreas volume (cm^3)												Fat/ Parenchyma ratio		
		Total				Parenchyma				Fat						
**Total subjects**	**135**															
0 - 2.5	30	8.9	±	5.7		6.0	±	3.9		2.9	±	2.0		0.49	±	0.20
2.5 - 5	16	20.1	±	5.7	**	14.5	±	3.9	**	5.6	±	2.2		0.39	±	0.11
5 - 10	27	28.7	±	7.6	*	20.3	±	5.1	*	8.4	±	4.0		0.42	±	0.17
10 - 15	29	45.7	±	12.8	**	32.1	±	8.0	**	13.6	±	6.1	**	0.42	±	0.14
15 - 20	33	64.1	±	18.0	**	43.1	±	11.8	**	21.0	±	9.3	**	0.50	±	0.21
**Male**	**77**															
0 - 2.5	18	9.7	±	6.4		6.5	±	4.3		3.2	±	2.3		0.51	±	0.18
2.5 - 5	8	19.9	±	6.6	*	13.9	±	4.3	*	6.0	±	2.6		0.43	±	0.12
5 - 10	17	27.4	±	7.0		19.2	±	4.0		8.3	±	4.2		0.43	±	0.18
10 - 15	17	47.4	±	12.5	**	33.6	±	7.8	**	13.8	±	6.2	**	0.41	±	0.13
15 - 20	17	68.0	±	16.9	**	44.5	±	9.6	**	23.5	±	10.0	**	0.53	±	0.19
**Female**	**58**															
0 - 2.5	12	7.6	±	4.3		5.3	±	3.2		2.3	±	1.4		0.46	±	0.23
2.5 - 5	8	20.2	±	5.0	*	15.1	±	3.7	*	5.2	±	1.7		0.34	±	0.08
5 - 10	10	31.0	±	8.5		22.2	±	6.4		8.8	±	3.6		0.41	±	0.18
10 - 15	12	43.3	±	13.5	*	30.0	±	8.2	*	13.3	±	6.2		0.44	±	0.16
15 - 20	16	60.0	±	18.7	**	41.6	±	13.9	**	18.4	±	7.9	*	0.46	±	0.23

Values are means ± SD. * $p < 0.05$, ** $p < 0.01$ vs. younger population.

<u>Supplementary Table 2.</u> Effects of Age on Pancreas Volume in Adulthood

Age (years)	n	BMI (kg/m^2)		Pancreas volume (cm^3)						Fat/ Parenchyma ratio	
				Total		Parenchyma		Fat			
**Total subjects**	**1721**										
20 - 29	80	24.0 ± 5.0		69.8 ± 21.4		49.6 ± 14.9		20.2 ± 11.4		0.42 ± 0.26	
30 - 39	142	25.4 ± 5.1	*	77.6 ± 23.5	*	51.5 ± 15.3		26.1 ± 14.0	**	0.54 ± 0.31	*
40 - 49	282	25.9 ± 5.4	**	77.8 ± 25.0	*	49.5 ± 16.5		28.3 ± 15.1	**	0.62 ± 0.35	**
50 - 59	430	25.6 ± 4.5	**	75.0 ± 26.4		47.3 ± 16.6	¶	27.8 ± 15.4	**	0.63 ± 0.36	**§
60 - 69	364	26.0 ± 5.0	**	72.4 ± 26.2	§†	42.7 ± 15.5	**¶†∬	29.7 ± 16.2	**§	0.74 ± 0.41	**¶†
70 - 79	277	25.0 ± 4.5	†	67.0 ± 26.2	¶†∬	38.2 ± 14.7	**¶†∬	28.8 ± 17.2	**	0.82 ± 0.55	**¶†∬
80 - 89	131	24.0 ± 3.9	§†∬	60.6 ± 23.1	*¶†∬	33.1 ± 12.8	**¶†∬	27.5 ± 16.3	**	0.91 ± 0.61	**¶†∬
90-	15	22.9 ± 3.6	†∫	58.7 ± 15.2	¶†∫	32.8 ± 13.5	**¶†∬	25.9 ± 11.3		0.98 ± 0.63	**¶†∬
**Male**	**724**										
20 - 29	37	24.5 ± 4.4		75.8 ± 24.7		53.9 ± 17.2		21.9 ± 12.5		0.42 ± 0.24	
30 - 39	57	27.0 ± 4.3	**	85.8 ± 23.5		56.4 ± 14.9		29.4 ± 14.2	*	0.54 ± 0.28	
40 - 49	99	26.9 ± 4.2	**	93.5 ± 26.9	**	54.9 ± 17.5		38.5 ± 17.5	**¶	0.76 ± 0.39	**¶
50 - 59	179	26.0 ± 3.9	*	88.2 ± 28.6	**	51.6 ± 18.0		36.6 ± 16.8	**¶	0.76 ± 0.35	**¶
60 - 69	163	26.9 ± 4.7	**	87.0 ± 25.2	*	47.8 ± 16.2	*¶†∫	39.2 ± 16.1	**¶	0.90 ± 0.42	**¶†∬
70 - 79	123	25.3 ± 3.6	¶†	83.1 ± 27.4	†	43.8 ± 15.9	**¶†∬	39.3 ± 18.7	**¶	0.98 ± 0.53	**¶†∬
80 - 89	58	24.2 ± 3.4	¶†∬	70.1 ± 21.3	¶†∬	35.6 ± 13.1	**¶†∬	34.5 ± 15.7	**	1.09 ± 0.65	**¶†∬
90-	8	22.4 ± 1.6	¶†∫	62.2 ± 16.2	§†∬	30.3 ± 14.1	**¶†∬	31.9 ± 9.2		1.28 ± 0.62	**¶†∬
**Female**	**997**										
20 - 29	43	23.5 ± 5.4		64.6 ± 16.6		45.9 ± 11.6		18.7 ± 10.3		0.43 ± 0.27	
30 - 39	85	24.3 ± 5.3		72.1 ± 22.0	*	48.3 ± 14.7		23.8 ± 13.5		0.53 ± 0.33	
40 - 49	183	25.4 ± 5.9		69.4 ± 19.3		46.6 ± 15.3		22.8 ± 10.0		0.54 ± 0.30	
50 - 59	251	25.4 ± 4.8		65.6 ± 20.0	¶†	44.2 ± 14.8	§	21.5 ± 10.5		0.53 ± 0.33	
60 - 69	201	25.2 ± 5.1		60.5 ± 20.5	¶†∬	38.6 ± 13.7	**¶†∬	21.9 ± 11.5		0.61 ± 0.34	**∫
70 - 79	154	24.8 ± 5.1		54.1 ± 16.4	**¶†∬	33.7 ± 12.0	**¶†∬	20.4 ± 9.7		0.70 ± 0.53	**¶†∬
80 - 89	73	23.9 ± 4.3		53.0 ± 21.7	**¶†∬	31.1 ± 12.3	**¶†∬	22.0 ± 14.6		0.77 ± 0.54	**¶†∬
90-	7	23.5 ± 5.2		54.7 ± 14.1	§	35.7 ± 13.2	§†	19.0 ± 9.9		0.63 ± 0.47	

Values are means ± SD.

* $p < 0.05$, ** $p < 0.01$ vs. age 20-29.

§ $p < 0.05$, ¶ $p < 0.01$ vs. age 30-39.

† $p < 0.05$, † $p < 0.01$ vs. age 40-49.

∫ $p < 0.05$, ∬ $p < 0.01$ vs. age 50-59.

Supplementary Table 3. **Effects of BMI on Pancreas Volume in Adulthood**

	n	Age (years)	BMI (kg/m^2)	Pancreas volume (cm^3) Total	Parenchyma	Fat	Fat/ Parenchyma ratio
**Total subjects**	**1150**						
Lean	460	57 ± 14	22.1 ± 1.8	66.2 ± 22.0	43.2 ± 15.7	23.0 ± 12.4	0.59 ± 0.37
Overweight	460	57 ± 14	27.1 ± 1.4 **	76.6 ± 23.8 **	46.2 ± 16.2 **	30.4 ± 14.3 **	0.73 ± 0.45 **
Obese	230	57 ± 14	33.6 ± 3.4 **§§	87.3 ± 30.3 **§§	48.7 ± 18.0 **	38.6 ± 18.9 **§§	0.86 ± 0.44 **§§
**Male**	**495**						
Lean	198	56 ± 15	22.5 ± 1.8	77.0 ± 22.7	47.5 ± 16.3	29.5 ± 13.5	0.68 ± 0.36
Overweight	198	56 ± 15	27.1 ± 1.4 **	89.6 ± 23.4 **	50.7 ± 17.1	38.9 ± 14.6 **	0.85 ± 0.43 **
Obese	99	56 ± 15	33.3 ± 3.3 **§§	103.8 ± 30.9 **§§	54.7 ± 19.2 **	49.1 ± 20.5 **§§	0.99 ± 0.49 **§§
**Female**	**655**						
Lean	262	58 ± 13	21.8 ± 1.7	58.1 ± 17.4	39.9 ± 14.4	18.1 ± 8.8	0.52 ± 0.35
Overweight	262	59 ± 13	27.0 ± 1.4 **	66.7 ± 18.9 **	42.8 ± 14.6 *	23.9 ± 9.9 **	0.63 ± 0.45 **
Obese	131	58 ± 14	33.9 ± 3.4 **§§	74.8 ± 23.1 **§§	44.2 ± 15.7 **	30.7 ± 12.7 **§§	0.75 ± 0.37 **§§

Values are means ± SD. * $p < 0.05$, ** $p < 0.01$ vs. lean. § $p < 0.05$, §§ $p < 0.01$ vs. overweight.
